# Recurrent venous thromboembolism patients form clots with lower elastic modulus than those formed by patients with non‐recurrent disease

**DOI:** 10.1111/jth.14402

**Published:** 2019-03-08

**Authors:** Stephen R. Baker, Michal Zabczyk, Fraser L. Macrae, Cédric Duval, Anetta Undas, Robert A. S. Ariëns

**Affiliations:** ^1^ Leeds Thrombosis Collective Department of Discovery and Translational Science Leeds Institute of Cardiovascular and Metabolic Medicine University of Leeds Leeds UK; ^2^ Institute of Cardiology Jagiellonian University Medical College Krakow Poland; ^3^ John Paul II Hospital Krakow Poland

**Keywords:** fibrin, mechanics, recurrent event, rheology, venous thromboembolism

## Abstract

**Essentials**

- Venous thromboembolism (VTE) recurrence leads to decreased clot elastic modulus in plasma.
- Recurrent VTE is not linked to changes in clot structure, fiber radius, or factor XIII activity.
- Other plasma components may play a role in VTE recurrence.
- Prospective studies should resolve if clot stiffness can be used as predictor for recurrent VTE.

**Summary:**

**Background**

Venous thromboembolism (VTE) is associated with a high risk of recurrent events after withdrawal of anticoagulation.

**Objectives**

To determine the difference in plasma clot mechanical properties between patients with recurrent VTE (rVTE) and those with non‐recurrent VTE (nrVTE).

**Methods**

We previously developed a system for determining clot mechanical properties by use of an in‐house magnetic tweezers system. This system was used to determine the mechanical properties of clots made from plasma of 11 patients with rVTE and 33 with nrVTE. Plasma was mixed with micrometer‐sized beads, and thrombin and calcium were added to induce clotting; the mixture was then placed in small capillary tubes, and clotting was allowed to proceed overnight. Bead displacements upon manipulation with magnetic forces were analyzed to determine clot elastic and viscous moduli. Fibrin clot structure was analyzed with turbidimetry and confocal microscopy. Factor XIII was measured by pentylamine incorporation into fibrin.

**Results**

Clots from rVTE patients showed nearly two‐fold less elastic and less viscous moduli than clots from nrVTE patients, regardless of male sex, unprovoked events, family history of VTE, fibrinogen concentration, or body mass index. No differences were observed in clot structure, fibrinolysis rates, or FXIII levels.

**Conclusion**

Using magnetic tweezers for the first time in patient samples, we found that plasma clots from rVTE patients showed a reduced elastic modulus and a reduced viscous modulus as compared with clots from nrVTE patients. These data indicate a possible role for fibrin clot viscoelastic properties in determining VTE recurrence.

## Introduction

Venous thromboembolism (VTE), which encompasses deep vein thrombosis (DVT) and pulmonary embolism (PE), affects 0.1–0.3% of people per year 1, 2. It is divided into two different categories, i.e. provoked and unprovoked (idiopathic); the latter is characterized by the patient having no history of cancer, surgery requiring general anesthesia, major trauma, plaster cast or recent hospitalization, including for pregnancy or delivery. VTE treatment is aimed at preventing and predicting recurrence, which may lead to death, thromboembolic pulmonary hypertension, and post‐thrombotic syndrome 3, 4, 5.

Studies in recent years have indicated that fibrin clot properties constitute a novel risk factor for both DVT and PE 6. Clots from these patients have been shown to have thinner, more highly branched fibers, and are less porous and more resistant to lysis than clots from healthy controls. These clot abnormalities persist or worsen in patients with post‐thrombotic syndrome 7. Furthermore, circulating fibrinogen levels have also been shown to increase in VTE patients 8. In addition, increases in clot lysis time have been shown to be weakly associated with VTE recurrence in women but not in men 9. Mechanically, clots made from PE patient plasma have been shown to develop viscoelastic properties at an accelerated rate as compared with clots made from DVT patients, although, interestingly, the final mechanical properties for these two patient groups were similar 10.

In this study, we used magnetic microrheology for the first time to determine the viscoelastic properties of plasma clots from patients with recurrent VTE (rVTE) and non‐recurrent VTE (nrVTE). Magnetic microrheology is advantageous in that it is much more sensitive to small changes than traditional rheology experiments, and it requires only low plasma volumes of 30 μL per patient. Previous studies using other methods for the analysis of clot mechanical properties required higher patient plasma volumes, ranging from 100 μL 10 to as much as 4.5 mL 11. We found significant differences in plasma clot viscoelastic properties between patients with rVTE and patients with nrVTE. These data suggest that this technique may be a useful diagnostic tool for determining patient clot elastic modulus as a novel risk factor for VTE recurrence.

## Materials and methods

### VTE patients

Forty‐four patients were recruited to the study: 33 patients who had experienced one first‐ever‐documented VTE episode, and 11 patients who had experienced two or more VTE episodes. All patients were aged between 18 years and 63 years (median, 34 years). Patients were eligible if at least 3 months had elapsed from their last VTE episode. The diagnosis of DVT of the lower or upper limb was established by a positive finding on color duplex sonography (visualization of an intraluminal thrombus in the calf, popliteal, femoral, or iliac vein). The diagnosis of PE was based on the presence of typical symptoms and positive results on high‐resolution spiral computed tomography. An unprovoked (idiopathic) VTE episode was defined as the patient having no history of cancer, surgery requiring general anesthesia, major trauma, plaster cast or hospitalization in the last month, or pregnancy or delivery in the last 3 months. A family history of VTE was defined as the patient having at least one first‐degree relative with VTE. The exclusion criteria were: acute infection, known malignancy, acute coronary syndromes or stroke within the preceding 6 months, indication for long‐term anticoagulation for reasons than VTE, end‐stage kidney disease, inherited and acquired thrombophilias, including factor V Leiden mutation and prothrombin G20210A, deficiencies in natural anticoagulants, i.e. antithrombin, protein C, or protein S, and refusal to provide consent. The Jagiellonian University Ethical Committee approved the study, and participants provided informed consent in accordance with the Declaration of Helsinki.

### Blood collection and analysis

Blood samples were taken from the antecubital vein at least 3 months after the patient's last VTE, between 8 a.m. and 11 a.m. For patients taking low molecular weight heparin, blood was collected 20–24 h after their last injection. For patients receiving vitamin K antagonists or rivaroxaban, blood was collected at least 24 h after their last drug intake. All other samples were collected at least 3–5 months after the last anticoagulant withdrawal. Smokers were asked to refrain from smoking for 2 h prior to blood collection. Blood samples (9 : 1 v/v 3.2% trisodium citrate) were centrifuged at 2000 × *g* for 10 min, and the supernatant was aliquoted and stored at − 80 °C for analysis. Routine laboratory assays were used to determine basic biochemical parameters. The fibrinogen concentration was determined with the Clauss method.

Fibrinogen was purified from 16 VTE patients (five with rVTE; 11 with nrVTE) by the use of IF1 (fibrinogen mAb; Kamiya Biomedical Company, Seattle, WA, USA) affinity chromatography as described previously 12. Purified fibrinogen was subjected to 10% SDS‐PAGE to confirm purity and the absence of protein degradation. Isolated fibrinogen was pooled to obtain a concentration of 1.0 g L^−1^. Finally, it was suspended in Tris‐buffered saline (0.1 mol L^−1^ Tris‐HCl and 0.154 mol L^−1^ NaCl, pH 7.4).

All measurements were performed by researchers who were blinded to the origin of the samples.

### Magnetic microrheology

Viscoelastic properties were measured as previously described 13, 14. Plasma samples were diluted 1 : 10 (final volume) in Tris‐buffered saline (TBS) (50 mmol L^−1^ Tris‐HCl and 100 mmol L^−1^ NaCl_2_, pH 7.4) and mixed with superparamagnetic beads at 1 : 250 (v/v) (4.5 μm in diameter, Dynabeads M‐450 Epoxy; Invitrogen, Paisley, UK). To induce clotting, an activation mixture of 0.5 U mL^−1^ human α‐thrombin (Merck, Millipore, Watford, UK) and 5 mmol L^−1^ CaCl_2_ was added to the plasma and quickly pipette‐mixed. Immediately after mixing, the solution was pulled into a capillary tube (CM Scientific Ltd, Keighley, UK), sealed on both ends with petroleum jelly, and allowed to clot overnight. For purified samples, final concentrations of 0.5 mg mL^−1^ fibrinogen, 0.5 U mL^−1^ thrombin, and 2.5 mmol L^−1^ CaCl_2_ were used for all experiments.

Clot viscoelasticity was measured by suspending the capillary between four electromagnets on top of an inverted microscope (Olympus IX71, Southend‐on‐sea, UK). A long‐working‐distance × 40 objective and CCD camera were used to locate the beads suspended within each clot. Bead tracking, image analysis and control of the electromagnets were performed with custom labview software (National Instruments, Newbury, UK). The elastic (*G*ʹ) and the viscous (*G*″) shear modulus were calculated from the time‐dependent compliance (ratio of time‐dependent shear strain or bead displacement to the magnitude of the applied force), which allows for determination of frequency‐dependent moduli. The loss tangent (tan*δ* = *G*″/*G*ʹ) was calculated as the ratio between the viscous and elastic moduli. More viscous materials result in a tan*δ* that is > 1. More elastic materials result in a tan*δ* that is < 1. For a completely viscoelastic material, tan*δ* will be equal to 1. The displacement of 10 random beads was measured per sample.

### Turbidimetric analysis of fiber radius and internal structure

Average fiber radius and average number of protofibrils per fiber were determined with turbidimetric measurements as previously described 15, taking into account that the index of refraction changes as a function of wavelength, as described by Ferri *et al*. 16. Clots were allowed to form in UV–visible transparent plastic cuvettes (Eppendorf UVette; Sigma‐Aldrich, Dorset, UK) overnight in a final volume of 100 μL. Plasma was diluted 1 : 6 (final volume) in TBS, and clots were activated with final concentrations of 0.5 U mL^−1^ human α‐thrombin and 5 mmol L^−1^ CaCl_2_. Plasma was not available from two rVTE and three nrVTE patients for these experiments. Purified samples were diluted to final concentrations of 0.5 mg mL^−1^ fibrinogen, and activated with final concentrations of 0.5 U mL^−1^ human α‐thrombin and 2.5 mmol L^−1^ CaCl_2_. A purified sample was not available from one rVTE patient for these experiments. The cuvette was sealed with parafilm to prevent dehydration. After clot formation, the cuvette was scanned over wavelengths between 500 < *λ* < 800 nm in a *Λ* 35 UV–visible spectrophotometer (Perkin‐Elmer, Cambridge, UK). Measurements for each patient (plasma or purified) were performed in triplicate.

### Turbidity and fibrinolysis

Clot formation and fibrinolysis were measured in 96‐well plates (Greiner, Stonehouse, UK) with a Powerwave microplate reader (Bio‐Tek, Swindon, UK) as previously described 17. Plasma was diluted to a final concentration of 1 : 6 in TBS. Clot formation for turbidity and fibrinolysis measurements was activated by adding final concentrations of 0.1 U mL^−1^ human α‐thrombin and 10 mmol L^−1^ CaCl_2_. For fibrinolysis measurements, tissue‐type plasminogen activator was added at a final concentration of 30 ng mL^−1^. The absorbance (or OD) was measured at a wavelength of 340 nm every 12 s for 3.5 h at 37 °C. Absorbance for turbidity and fibrinolysis measurements was determined in duplicate for all plasma samples. Turbidity profiles were analyzed for maximum absorbance (maximum OD), lag time, time to maximum absorbance, and average rate of clot formation. Fibrinolysis was analyzed for average rate of fibrinolysis and time to half‐maximum absorbance (half lysis).

### Laser scanning confocal microscopy

Structural analysis of VTE patient plasma clots was performed with laser scanning confocal microscopy. Plasma was diluted to a final volume of 1 : 4 in TBS, and spiked with Alexa Fluor 488‐labeled fibrinogen at a final concentration of 50 μg mL^−1^. Clotting was initiated by adding human α‐thrombin and CaCl_2_ at final concentrations of 0.1 U mL^−1^ and 10 mmol L^−1^, respectively. Immediately following clot initiation, clotting solution was transferred to a well of an uncoated six‐well Ibidi slide (Ibidi GmbH, Martinsried, Germany), placed in a humidity chamber, and left to clot for 3 h at room temperature. Images were taken with a Zeiss LSM880 inverted microscope with a × 40 oil immersion objective lens (Carl Zeiss Ltd, Cambridge, UK). Three images were taken for each clot, and Z‐stacks (30 slices at 20‐μm total distance) were combined and flattened to show maximum intensity (imagej; NIH, Bethesda, MD, USA).

### 5‐(Biotinamido)pentylamine incorporation assay

Measurement of FXIII activation and activity was performed with a modified 5‐(biotinamido)pentylamine incorporation assay, as previously described 18. Nunc‐Immuno 96 MicroWell plates (Fisher Scientific, Loughborough, UK) were coated with 100 μL of 40 μg mL^−1^ FXIII‐free fibrinogen for 40 min at room temperature, and then blocked with 300 μL of 1% (w/v) bovine serum albumin (BSA) in TBS for 90 min at 37 °C. Plates were washed with 4 × 300 μL TBS, and 10 μL of sample, diluted 1 : 5 in TBS, was added to the wells. Ninety microliters of activation mix (111 μmol L^−1^ dithiothreitol, 0.3 μmol L^−1^ biotinylated pentylamine, 11 mmol L^−1^ CaCl_2_, and 2.2 U mL^−1^ thrombin) was added, and the reactions were stopped at 0, 5, 10, 15 and 120 min by addition of 200 μL of 200 mmol L^−1^ EDTA. Plates were washed with 4 × 300 μL of 0.1% (v/v) Tween 20 in TBS, and 100 μL of 2 μg mL^−1^ streptavidin in 1% (w/v) BSA (in TBS–Tween) was added for 60 min at 37 °C. Following washes with 4 × 300 μL of TBS–Tween, 100 μL of 1 mg mL^−1^ phosphatase substrate (in 1 mol L^−1^ diethanolamine) was added, and the reaction was stopped by addition of 100 μL of 4 mol L^−1^ NaOH. Absorbance was measured at 405 nm, with a SpectraMax 190 absorbance microtiter plate reader (Molecular Devices, Wokingham, UK). The rate of pentylamine incorporation over early time points was used as an indicator of FXIII activation, and the data from the final time point were used to quantify final FXIII activity.

### Statistical analysis

Categorical variables are presented as numbers and percentages. Continuous variables are expressed as mean ± standard deviation or median and interquartile range. Normality was assessed with the Shapiro–Wilk test. Differences between groups were analyzed by the use of Student's *t*‐test for normally distributed variables and the Mann–Whitney *U*‐test for non‐normally distributed continuous variables. Categorical variables were compared by use of the chi‐square test. All calculations were performed with statistica Version 12.5 (StatSoft, Tulsa, OK, USA).

## Results

### Patients with rVTE form clots with a decreased elastic modulus

**Figure 1:**
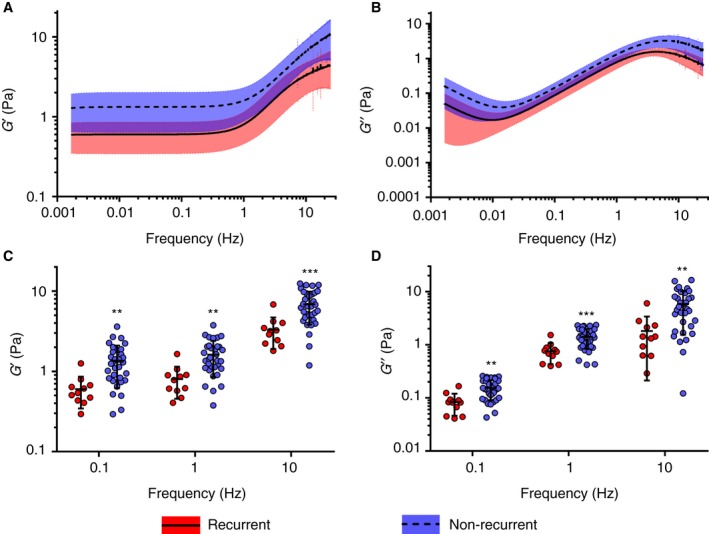
Viscoelastic moduli for clots made with platelet‐poor plasma from patients with recurrent or non‐recurrent venous thromboembolism (VTE). (A, B) Log–log plots of *G*ʹ or storage (elastic) modulus (A) and *G*″ or loss modulus (B) over all measured frequencies. (C, D) Comparison of storage modulus (C) and loss modulus (D) corresponding to specific relaxation modes (0.1, 1 and 10 Hz) showed that, at all frequencies, non‐recurrent VTE samples had significantly higher modulus values that recurrent VTE samples. **P < 0.01, ***P < 0.001.

The demographic characteristics of the patients, C‐reactive protein, fibrinogen, anticoagulation and International Normalized Ratio are shown in Table 1. The mechanical properties of plasma clots from patients with rVTE (*n* = 11) showed a reduced elastic modulus as compared with those from patients with nrVTE (*n* = 33) over all frequencies measured (Fig. 1A). Specific frequency values corresponding to different shear relaxation modes (0.1, 1 and 10 Hz) were used to study clot mechanical behavior 19. These frequencies are shown in Fig. 1C. There were significant increases in storage modulus (*G*ʹ) at 0.1 Hz (1.30 Pa versus 0.54 Pa), 1 Hz (1.43 Pa versus 0.78 Pa) and 10 Hz (6.49 Pa versus 3.18 Pa) for nrVTE versus rVTE (Table 2). There was also a significant increase in loss modulus (*G*″) over each of these frequencies (0.15 Pa versus 0.08 Pa at 0.1 Hz, 1.37 Pa versus 0.74 Pa at 1 Hz, and 4.69 Pa versus 1.43 Pa at 10 Hz; Fig. 1B,D; Table 2). The loss tangent (tan*δ* = *G*″/*G*ʹ), which is a measure of the relative ratio between viscous and elastic properties, showed no difference at either 0.1, 1 or 10 Hz (Table 2).

**Table 1:** Characteristics of patients with recurrent and non‐recurrent venous thomboembolism (VTE)

Variable	VTE patients (*n* = 44)	Recurrent VTE (*n* = 11)	Non‐recurrent VTE (*n* = 33)	*P*‐value
Age (years), median (25–75% quartile)	34 (32–47)	34 (32–40)	35 (29–48)	0.87
Male sex, *n* (%)	13 (28.9)	5 (45.5)	8 (23.5)	0.16
Body mass index (kg/m^2^), mean ± SD	27.0 ± 5.4	25.2 ± 5.7	26.8 ± 5.4	0.99
DVT alone, *n* (%)	15 (34.1)	4 (36.4)	11 (33.3)	0.85
PE alone, *n* (%)	15 (34.1)	4 (36.4)	11 (33.3)	0.85
Unprovoked VTE, *n* (%)	25 (55.6)	9 (81.8)	16 (47.1)	0.044
Family history of VTE, *n* (%)	7 (15.6)	0	7 (20.6)	0.12
Time since the index event (months), median (25–75% quartile)	12 (8–22)	12 (8–18)	14 (8–24)	0.95
White blood cells (10^3^ μL^–1^), mean ± SD	6.11 ± 1.52	6.0 ± 1.32	6.14 ± 1.59	0.78
Red blood cells (10^6^ μL^–1^), mean ± SD	4.76 ± 0.41	4.81 ± 0.47	4.74 ± 0.39	0.52
Haemoglobin (g dL^–1^), mean ± SD	13.9 ± 1.4	14.2 ± 1.2	13.8 ± 1.5	0.52
Hematocrit (%), mean ± SD	41.5 ± 3.3	42.6 ± 3.3	41.2 ± 3.3	0.23
Red cell distribution width (%), mean ± SD	13.3 ± 0.9	13.6 ± 1.2	13.2 ± 0.8	0.36
Platelets (10^3^ μL^–1^), mean ± SD	246 ± 65	244 ± 83	246 ± 60	0.96
Glucose (mmol L^−1^), median (25–75% quartile)	5.1 (4.8–5.4)	5.0 (4.7–5.3)	5.1 (4.8–5.6)	0.70
Total cholesterol (mmol L^−1^), mean ± SD	5.22 ± 1.20	5.86 ± 1.25	5.01 ± 1.12	0.07
LDL cholesterol (mmol L^−1^), mean ± SD	3.44 ± 1.06	4.02 ± 1.05	3.26 ± 1.0	0.038
HDL cholesterol (mmol L^−1^), mean ± SD	1.48 ± 0.33	1.48 ± 0.35	1.47 ± 0.32	0.81
Triglycerides (mmol L^−1^), median (25–75% quartile)	1.23 (0.86–1.77)	1.30 (0.97–1.83)	1.21 (0.83–1.68)	0.48
C‐reactive protein (mg L^–1^), median (25–75% quartile)	1.50 (0.84–4.25)	2.37 (1.04–6.38)	1.50 (0.83–2.77)	0.25
D‐dimer (ng mL^–1^), median (25–75% quartile)	247 (171–406)	250 (170–435)	278 (171–384)	0.95
Fibrinogen (g L^–1^), median (25–75% quartile)	3.11 ± 0.62	3.01 ± 0.61	3.14 ± 0.63	0.61
Vitamin K antagonist, *n* (%)	19 (43.2)	4 (36.4)	15 (45.5)	0.74
INR, median (25–75% quartile)	1.06 (0.99–1.51)	1.03 (1.00–1.45)	1.07 (0.99–1.61)	0.91
Rivaroxaban, *n* (%)	4 (9.1)	0 (0)	4 (12.1)	0.56
LMWH, *n* (%)*	3 (6.8)	2 (18.2)	1 (3.0)	0.14

DVT, deep vein thrombosis; INR, International Normalized Ratio; LMWH, low molecular weight heparin; PE, pulmonary embolism; SD, standard deviation. *P*‐values were obtained with the *χ* ^2^ test for sex, unprovoked VTE, family history of VTE, vitamin K antagonist, rivaroxaban, and LMWH, and with Student's *t*‐test for body mass index and fibrinogen. All other *P*‐values were obtained with a Mann–Whitney test. *For the three samples from patients receiving LMWH, the level of anti factor Xa was determined to be < 0.02 IU mL^–1^ for each sample.

**Table 2:** Viscoelastic properties of venous thromboembolism (VTE) plasma clots

	VTE patients (*n* = 44)	Recurrent VTE (*n* = 11)	Non‐recurrent VTE (*n* = 33)	*P*‐value
*G*′ 0.1 Hz (Pa)	0.98 (0.62–0.50)	0.54 (0.43–0.67)	1.30 (0.80–1.81)	0.002
*G*′ 1 Hz (Pa)	1.24 (0.86–1.94)	0.78 (0.57–0.90)	1.43 (1.10–2.04)	0.0015
*G*′ 10 Hz (Pa)	5.29 (3.54–8.50)	3.18 (2.22–4.02)	6.49 (4.53–9.49)	0.0006
*G*″ 0.1 Hz (Pa)	0.13 (0.08–0.18)	0.08 (0.05–0.09)	0.15 (0.10–0.21)	0.0011
*G*″ 1 Hz (Pa)	1.16 (0.78–1.67)	0.74 (0.43–0.88)	1.37 (0.94–1.95)	0.0008
*G*″ 10 Hz (Pa)	3.86 (1.43–7.45)	1.43 (0.63–2.31)	4.69 (2.01–8.75)	0.0043
Tan*δ* 0.1 Hz	0.12 (0.10–0.15)	0.13 (0.11–0.18)	0.11 (0.10–0.15)	0.43
Tan*δ* 1 Hz	0.90 (0.78–1.02)	0.95 (0.83–1.03)	0.89 (0.77–1.02)	0.53
Tan*δ* 10 Hz	0.59 (0.32–1.16)	0.39 (0.28–0.66)	0.64 (0.44–1.21)	0.16

Values are given as median (25–75% quartile). *P*‐values were obtained with the Mann–Whitney test.

Fibrinogen was purified from a subset of these plasma samples. In contrast to what we found for plasma clots, we found no difference in viscoelastic properties (*G*ʹ or *G*″) between rVTE samples (*n* = 6) and nrVTE samples (*n* = 11) (Fig. S1).

### Fiber diameter and intrafibrillar structure

**Figure 2:**
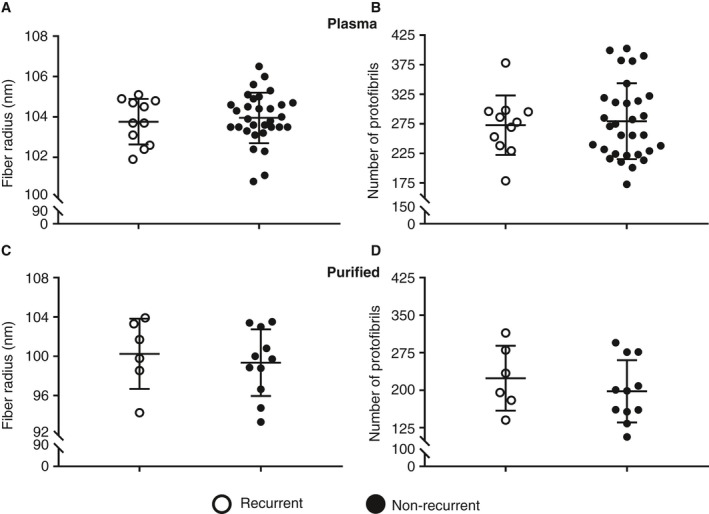
Average fiber radius and protofibril number for purified and plasma clots from recurrent and non‐recurrent venous thromboembolism (VTE) patients. No differences in average fiber radius or average number of protofibrils per fibrin fiber were observed between recurrent and non‐recurrent VTE in either plasma (A, B) or purified fibrin (C, D) clots.

No significant differences were found in average fiber diameter or average number of protofibrils packed per fibrin fiber between plasma clots made from rVTE samples and those made from nrVTE samples, as shown in Fig. 2A,B. Similarly, clots made from purified fibrinogen showed no significant difference in either average fiber diameter or average number of protofibrils between rVTE and nrVTE samples (Fig. 2C,D).

### Plasma clot structure, formation, and fibrinolysis

**Figure 3:**
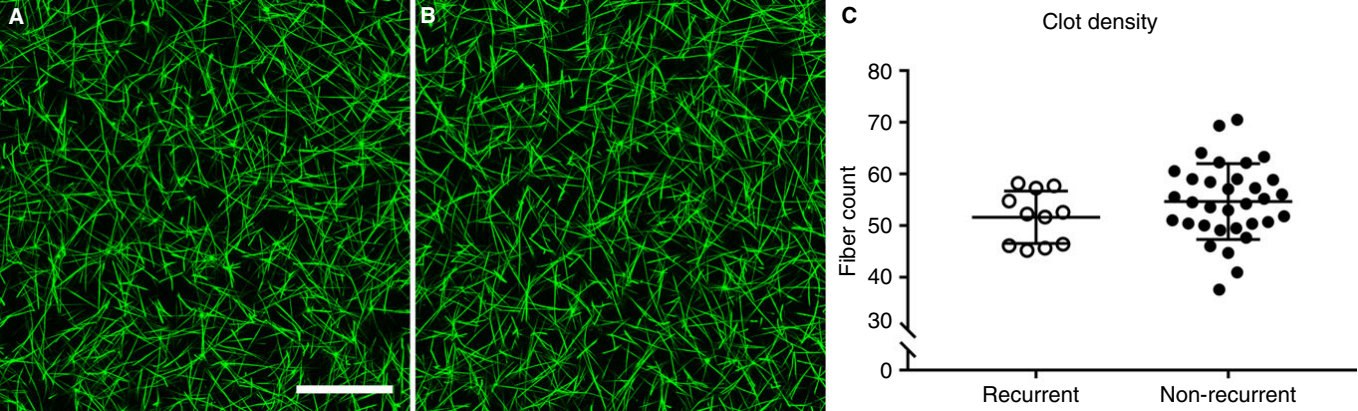
Comparison of plasma clot structure by the use of confocal microscopy. (A, B) Representative confocal images for recurrent (A) and non‐recurrent (B) plasma clots. Images are represented as flattened Z‐stacks at maximum intensity. Scale bar: 50 μm. (C) Average clot density measured by counting fibers along 10 100‐μm long, evenly spaced vertical and horizontal lines.

The structure of fully formed clots was studied with laser scanning confocal microscopy. No significant differences in clot structure were observed between plasma clots formed from rVTE samples (Fig. 3A) and those formed from nrVTE samples (Fig. 3B). Furthermore, there was no difference in the average number of fibers per 100 μm between rVTE (52 ± 5) and nrVTE (55 ± 7) plasma samples (Fig. 3C).

**Figure 4:**
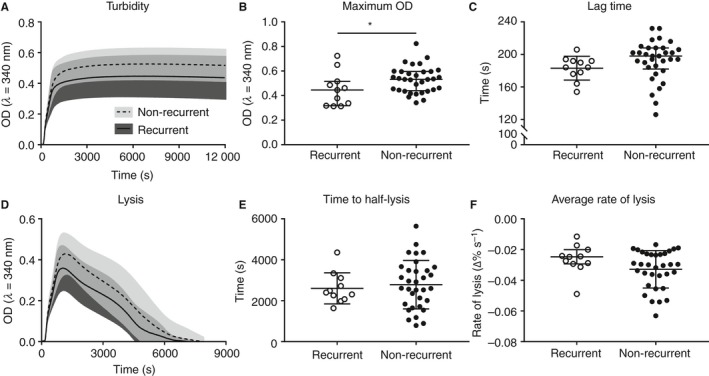
Turbidity and lysis for plasma clots from venous thromboembolism (VTE) patients. (A) Turbidity profile for non‐recurrent and recurrent VTE plasma clots measured at 340 nm. The line represents the mean, and the shaded area represents the standard deviation. (B) Maximum absorbance showed a significant difference between non‐recurrent and recurrent VTE samples. (C) Lag time or the amount of time to measurable absorbance showed no difference between samples. (D) Lysis profile for non‐recurrent and recurrent VTE plasma clots measured at 340 nm. (E, F) Time to half‐lysis (E) and average lysis rate (normalized change per second) (F) for plasma clots showed no difference between samples. *P < 0.05.

Next, we determined the average change in absorbance over time in plasma samples from rVTE and nrVTE patients (Fig. 4A). A significant decrease in maximum absorbance was observed in samples from rVTE patients (0.44 ± 0.14 at *λ* = 340 nm) as compared with samples from nrVTE patients (0.52 ± 0.11) (Fig. 4B). We found no significant differences in other clot formation properties observed, including: lag time (183 ± 15 s versus 193 ± 24 s; Fig. 4C), time to maximum absorbance (101 ± 20 s versus 110 ± 18 s), or average rate of clotting (0.12 ± 0.03 versus 0.12 ± 0.04, ∆OD s^–1^) (Table 3). Clots also broke down similarly over time by fibrinolysis (Fig. 4D). No significant differences were observed in either time to half‐lysis (44 ± 12 min versus 47 ± 20 min) or average rate of lysis (0.027 ± 0.010 versus 0.033 ± 0.012, ∆OD s^–1^) (Fig. 4E,F; Table 3). Note that all of the above values are given as rVTE versus nrVTE.

**Table 3:** Turbidity and lysis for venous thromboembolism (VTE) plasma clots

	VTE patients (*n* = 44)	Recurrent VTE (*n* = 11)	Non‐recurrent VTE (*n* = 33)	*P*‐value
Lag time (s)	190.1 ± 22.5	183.1 ± 14.6	192.5 ± 24.3	0.054
Time to maximum absorbance (min)	108 ± 19	101 ± 20	110 ± 18	0.170
Average rate of clotting (change OD s^–1^)	0.12 ± 0.04	0.12 ± 0.03	0.12 ± 0.04	0.730
Maximum absorbance (*λ* = 340 nm)	0.51 ± 0.12	0.44 ± 0.14	0.52 ± 0.11	0.046
Time to half‐lysis (min)	45.7 ± 18.1	43.5 ± 12.7	46.5 ± 19.8	0.642
Average rate of lysis (ΔOD s^–1^)	0.031 ± 0.012	0.027 ± 0.010	0.033 ± 0.012	0.122

Values are given as mean ± standard deviation. *P*‐values were obtained with Student's *t*‐test.

### FXIII activity

We measured FXIII activity for all patient plasma samples. The FXIII activation rate for rVTE samples (102% ± 11% of normal pooled plasma) was not different from that for nrVTE samples (107% ± 12%) (Fig. S2A). Similarly, the final FXIII activities for rVTE (102% ± 5% of normal pooled plasma) and nrVTE (98 ± 5%) plasma samples were unchanged (Fig. S2B).

## Discussion

Rheology is an important tool for studying the viscoelastic properties of materials, and has been widely used to study biological samples and, in particular, fibrin clots 20, 21, 22. The majority of rheology experiments use mechanical bulk rheometers to deform materials to differing strains 23. Traditional rheology experiments can be difficult to use on biological samples, such as plasma, because they typically require large volumes, are less sensitive to low viscosities, or are limited to low frequency ranges. Microrheology allows for a wide range of frequencies and can be carried out with very small sample volumes 23. As a result, microrheology is ideal for patient analysis when sample volumes can be limited.

To our knowledge, this is the first study to use a magnetic tweezers microrheology system to assess plasma fibrin viscoelastic properties in real‐life patient samples. We studied the mechanics of plasma and purified clots made from patients with rVTE and nrVTE. In plasma samples, nrVTE patients showed a reduced elastic modulus as compared with rVTE patients. In contrast, there were no significant differences in the mechanical properties (*G*ʹ or *G*″) of clots made from purified fibrinogen. As no difference in elastic modulus was observed for clots prepared from purified fibrinogen, the effects on clot mechanics in rVTE patients are therefore probably not caused by structural changes in fibrinogen itself. These data support the view that analysis of plasma clot features may provide more clinically relevant information on the association between clot phenotype and thromboembolic risk than purified fibrinogen samples.

Few studies have investigated the viscoelastic properties of clots made from VTE patients. Martinez *et al*. showed that, although there was accelerated establishment of viscoelastic properties for acute PE patients versus acute DVT patients, the final elastic and viscous properties were similar between the two groups 10. It should be noted that clots were not separated into rVTE or nrVTE in that particular study. In addition, these experiments were performed with a bulk rheometer, which, as noted above, is less sensitive to small changes in viscoelastic properties than microrheometry. The current study expands previous findings by showing altered viscoelastic clot characteristics in plasma from patients with rVTE versus plasma from patients with nrVTE, which highlights a potential use for fibrin viscoelastic assessment in the prediction of rVTE.

Interestingly, previous work has shown that clot structures for samples from patients with recurrent and non‐recurrent PE were different, with recurrent PE clots being more densely packed and less porous than non‐recurrent PE clots, although these clots were made with purified fibrinogen 24. Similarly, it was shown that recurrent DVT was associated with less permeable clots than non‐recurrent DVT, suggesting that these clots are less porous 25. In the current study, turbidity experiments showed that there was a higher maximum absorbance for nrVTE plasma samples. This could indicate that nrVTE plasma samples produce more densely packed clots. However, this could not be confirmed by analysis of clot architecture with laser scanning confocal microscopy, which showed no difference in fiber density between the groups.

Prolonged lysis times have been implicated as a risk factor for venous thrombosis 26. Some evidence suggests that increased lysis times predict recurrence of VTE in women but not in men 9, whereas other studies have suggested that increased lysis times predict the initial risk of venous thrombosis, but not the recurrence 26. Here, we found no difference in lysis time or rate of lysis between rVTE and nrVTE patient plasma samples, which is consistent with the absence of major changes in clot architecture in plasma clots from patients with rVTE.

Lower clot elastic moduli several months after the last VTE event may suggest that such properties enhance recurrence. It remains to be established whether a reduced elastic modulus of fibrin clots does enhance thrombus formation or embolization in prospective studies of patients with VTE.

As differences in viscoelastic properties were observed in plasma samples, but not in purified fibrinogen samples, we also analyzed typical markers for VTE recurrence (age, sex, body mass index, unprovoked VTE, and family history of VTE), although no significant differences were found. We observed no significant difference in either average fiber radius or number of protofibrils per fibrin fiber between plasma clots made from rVTE samples and those made from nrVTE samples. Similarly, clots made from purified fibrin showed no significant difference in either average fiber radius or average number of protofibrils between rVTE samples and nrVTE samples. It should be noted that the number of samples was limited and that the variation in these measurements was large. Finally, no changes in FXIII levels were observed, indicating that the change in elastic modulus for rVTE samples was not caused by changes in fibrin cross‐linking.

Predicting VTE recurrence from clot viscoelastic properties would allow for personalization of treatment for each individual patient. This might suggest that there could be multiple groups in the nrVTE samples, with one group having a lower clot elastic moduli than another. No significant grouping was observed within the nrVTE plasma samples, although there do seem to be specific samples with lower values than the average. Future prospective studies of clot mechanical characteristics in nrVTE patients, testing for recurrence, could help to determine whether clot elastic modulus can be used as a predictor for VTE recurrence.

In summary, we found a significantly reduced elastic modulus in plasma clots from patients with rVTE. The reduced elastic modulus was not associated with major changes in the elastic modulus of clots made from purified fibrinogen, plasma fibrinogen levels, plasma clot architecture, or plasma FXIII activity levels, suggesting that other plasma factors are at play in determining clot mechanics in patients with rVTE. Future studies in larger patient populations are required to corroborate these findings, elucidate the plasma‐based mechanisms involved, and test the prognostic value of clot elastic modulus in predicting VTE recurrence.

## Addendum

S. R. Baker conducted experiments, analyzed data, and wrote the manuscript. M. Zabczyk and A. Undas collected patient samples, purified fibrinogen, and edited the manuscript. F. L. Macrae and C. Duval conducted experiments, analyzed data, and edited the manuscript. R. A. S. Ariëns conceived the study, advised on experimental design and interpretation, and edited the manuscript.

## Disclosure of Conflict of Interests

The authors state that they have no conflict of interest.

## Supporting information


**Fig. S1.** Viscoelastic moduli for clots made with purified fibrin from patients with recurrent or non‐recurrent VTE.
**Fig. S2.** Plasma FXIII activity.
