# Supplementary material for: Recurrent venous thromboembolism patients form clots with lower elastic modulus than those formed by patients with non‐recurrent disease
Source: J Thromb Haemost. 2019 Mar 8;17(4):618–26. doi: 10.1111/jth.14402 (PMC6487944; doi:10.1111/jth.14402)
Supplement: Supplementary file 1 — Fig. S1. Viscoelastic moduli for clots made with purified fibrin from patients with recurrent or non‐recurrent VTE. Fig. S2. Plasma FXIII activity. [file JTH-17-618-s001.docx]

**
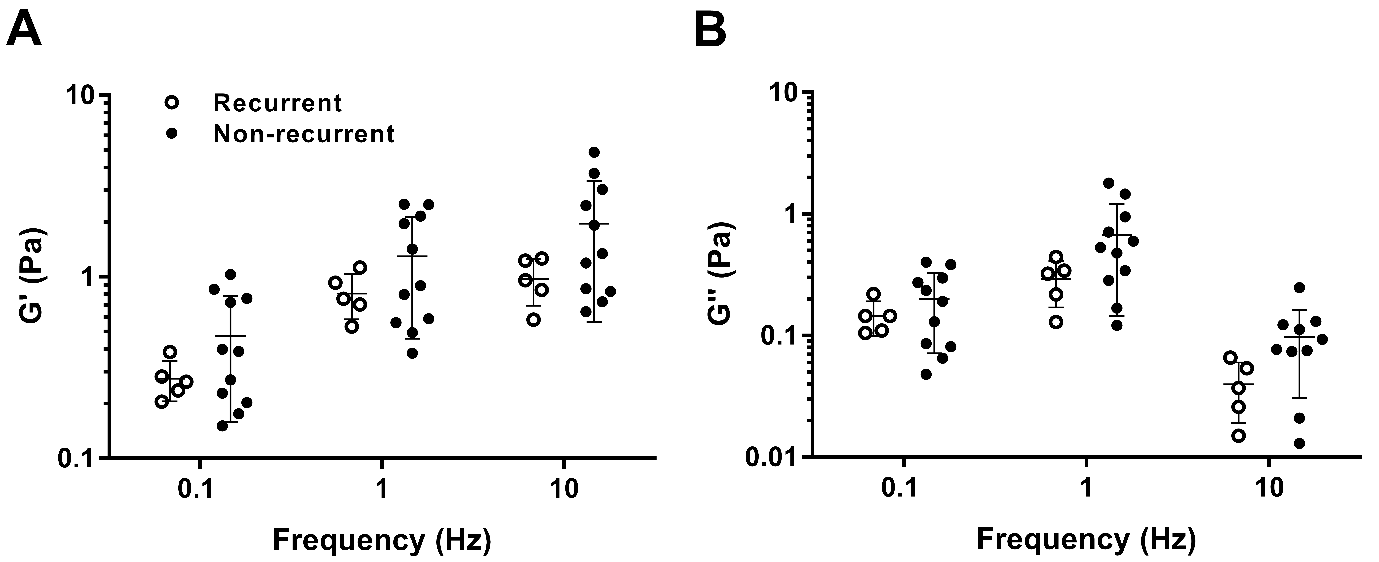
**

**Figure S1: Viscoelastic moduli for clots made with purified fibrin from patients with recurrent or non-recurrent VTE.** Comparison of G’ or storage (elastic) modulus (A) and G’’ or loss modulus (B) corresponding to specific relaxation modes (0.1, 1, 10 Hz).

**
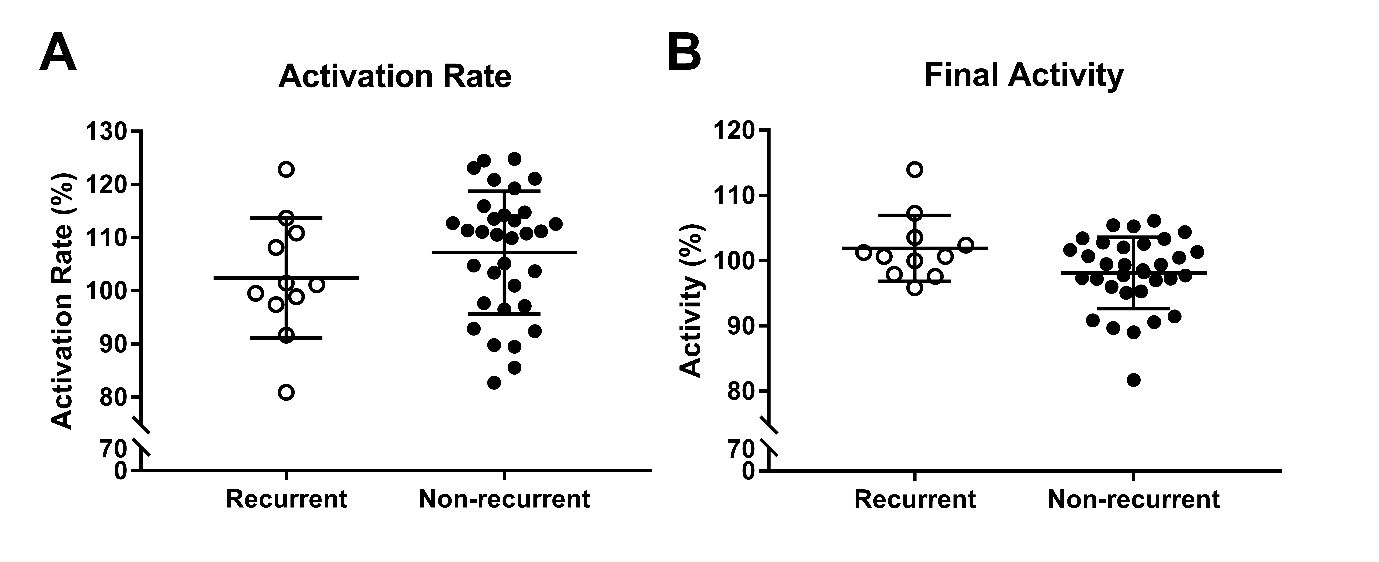
**

**Figure S2: Plasma FXIII activity.** FXIII activation rate for recurrent and non-recurrent patient samples (A). Final FXIII activity (B). Activation rate and activity are represented as percentage compared to activation rate and activity of normal pooled plasma.
